# Autophagy Stress Responses in Localized Prostate Cancer: A Flux-Aware Framework for Disease-Relevant Interpretation

**DOI:** 10.3390/cells15131134

**Published:** 2026-06-23

**Authors:** Zaira Edith Hernández-Ramírez, Enoc Mariano Cortés Malagón, Jonathan Puente-Rivera, Javier Flores-Estrada

**Affiliations:** 1 Research Division, Hospital Juárez de México, Mexico City 07760, Mexico; zairaedithhernandez@gmail.com (Z.E.H.-R.); emcortes@cinvestav.mx (E.M.C.M.); 2 Genetic Laboratory, Hospital Nacional Homeopático, Mexico City 06800, Mexico

**Keywords:** autophagy, stress response, autophagic flux, lysosomal competence, localized prostate cancer, proteostasis, mitophagy, selective autophagy, ferroptosis, disease models

## Abstract

Autophagy-associated readouts in localized prostate cancer cannot be interpreted based on LC3, p62/SQSTM1, or LC3 puncta alone. In line with the concept of autophagy as a stress-response system, this review proposes a flux-aware, organelle-centered framework for assigning biological meaning to autophagy-related changes under disease-relevant stress. The framework integrates oxidative burden, lysosomal competence, selective autophagy, mitophagy, ferritinophagy, p62/SQSTM1-NRF2 signaling, ferroptosis-aware controls, and disease-stage context to distinguish four interpretive states: homeostatic quality control, adaptive tumor survival, blocked clearance, and stress-overload vulnerability. Flavonoid-associated responses are used as stress-test examples because they expose recurrent limitations in the field, including supraphysiologic exposures, limited metabolite realism, static-marker inflation, and insufficient assessment of lysosomal function. However, the framework is not restricted to dietary compounds; it applies to metabolic, pharmacological, inflammatory, androgen-related, radiation-associated, or therapy-induced perturbations in which autophagy-associated markers are altered without resolution of flux or organelle function. By linking autophagosome formation, cargo turnover, lysosomal acidification, redox buffering, and phenotype-level endpoints, this review defines a practical evidence hierarchy for interpreting autophagy in localized prostate cancer and for prioritizing translational vulnerabilities arising from organelle crosstalk. This contribution is primarily conceptual and is operationalized methodologically through flux-based evaluation criteria and translationally through disease-window-specific study-design recommendations.

## 1. Introduction

Localized prostate cancer is an informative disease model for studying autophagy as a stress-response program because tissue exposure, lesion biology, redox tone, androgen signaling, lysosomal function, and short-term clinical trajectories can still be examined before metastatic selection and multi-line therapy obscure interpretation. In this clinical window, autophagy should be interpreted not as a simple marker of tumor suppression or tumor support, but as a dynamic organelle-centered process that can preserve homeostasis, support adaptation, reveal blocked clearance, or expose stress-overload vulnerability. This clinical setting is heterogeneous and includes patients managed through active surveillance or curative-intent approaches such as radical prostatectomy, external-beam radiotherapy, brachytherapy, and, depending on risk category, androgen-deprivation-based strategies. Radiotherapy is particularly relevant for a stress-response framework because localized low- and intermediate-risk disease can be treated with different fractionation schedules, and moderate hypofractionated radiotherapy has been described as a valid alternative strategy supported by randomized clinical evidence [1]. Reviews have addressed autophagy genes, autophagy in cancer, and prostate-cancer-specific autophagy contexts [2,3,4,5], whereas separate syntheses have discussed nutraceutical, soy/isoflavone, and flavonoid-associated activity in prostate cancer models [6,7,8]. Human intervention studies have also assessed green tea catechins, genistein, and polyphenol-rich interventions in localized or high-risk prostate settings [9,10,11,12,13,14]. However, most syntheses describe pathways and compounds more readily than they define when an autophagy-associated readout is biologically interpretable.

The central contribution of this review is therefore not another catalog of autophagy-modulating compounds. It is a state-based, flux-aware interpretive framework that treats autophagy as the organizing principle of a broader stress-response network in localized prostate cancer. Conceptually, the review reframes autophagy-associated readouts as disease-context-dependent states; methodologically, it defines the minimum assays needed to assign meaning to LC3, p62/SQSTM1, lysosomal, and redox signals; translationally, it links those states to clinically accessible windows such as HGPIN, active surveillance, and pre-prostatectomy studies [15,16,17].

That gap matters because autophagy in prostate cancer is not a directional biomarker. Depending on the androgenic context, nutrient availability, mitochondrial stress, and lysosomal function, the same machinery can limit oxidative damage and preserve tissue quality control, or maintain malignant fitness under hypoxia, therapy pressure, and metabolic strain [2,3,4,5,18,19,20,21]. A rigorous analysis must therefore decipher the biological state reflected in the readout rather than merely confirming that an autophagy-associated marker has changed.

Flavonoid-associated studies make this interpretive problem difficult to ignore. They are often presented as examples of antioxidants or autophagy-inducing chemoprevention. Yet, many reports rely on parent aglycones that do not reflect the conjugated species measured in human circulation and tissues, use concentrations unlikely to be reached in the prostate, or interpret LC3 and p62/SQSTM1 changes without flux resolution. Under these conditions, an apparently favorable signal may reflect adaptive redox buffering, non-canonical ATG8 lipidation, lysosomal congestion, or a broader organelle-stress program rather than productive macroautophagy.

Unlike previous reviews that discuss autophagy pathways or flavonoid activity as largely separate topics, this review addresses a broader disease-relevant problem in autophagy biology: when can an autophagy-associated readout generated under stress be assigned biological meaning in localized prostate cancer? Previous conceptual, prostate-focused, and autophagy methodology literature provides the background for this question [2,3,4,6,8,15,16,22]. We do not aim to rank flavonoids as therapeutic candidates. Instead, flavonoid-associated responses are used here as methodological probes within a broader stress-response framework. LC3, p62/SQSTM1, and puncta formation require flux-aware interpretation before they can be linked to productive autophagy [15,16,23]. ROS modulation may instead reflect adaptive buffering or stress amplification, depending on the biological context [24,25,26,27,28]. Lysosomal congestion and blocked clearance require lysosomal competence assays [29,30,31,32,33,34], whereas ferritinophagy-linked stress and organelle-overload claims require pathway-specific controls [35,36,37]. In this sense, flavonoids are illustrative perturbations within a broader stress-response framework that also includes metabolic stress, androgen-pathway manipulation, inflammatory cues, radiation, targeted therapy, and other disease-relevant conditions that alter autophagy-associated readouts without necessarily inducing productive degradation.

This positioning also clarifies the novelty relative to existing reviews of prostate cancer autophagy. Prior reviews have summarized autophagy machinery, therapeutic resistance, androgen signaling, and prostate cancer progression [2,3,17,38]. The present review builds on that literature but shifts the unit of interpretation from pathways or compounds to evidence-supported autophagy-associated states. The advance is therefore the integration of flux verification, lysosomal competence, redox burden, exposure realism, and localized-disease context into a single decision framework.

This framework directly addresses autophagy as a stress-response program by integrating oxidative burden and disease-stage context in localized prostate cancer [4,9,18,19,20,21,28]. It also incorporates lysosomal competence and selective autophagy as central interpretive determinants, because static changes in LC3 or p62/SQSTM1 cannot distinguish productive flux from blocked clearance without flux-aware validation [15,16,23] and assessment of lysosomal function [29,30,31,32,33]. In addition, mitophagy, ferritinophagy, p62/SQSTM1-NRF2 signaling, and ferroptosis-aware controls are included as stress-related modules that help classify whether the observed response reflects homeostatic control, adaptive tumor survival, blocked clearance, or stress-overload vulnerability. The p62/SQSTM1-NRF2 axis is supported mainly by redox-adaptation literature [19,20,39,40,41], whereas ferritinophagy and ferroptosis-aware interpretation require dedicated iron- and lipid-peroxidation controls [35,36,42,43,44].

This state-based framework is intended to improve both mechanistic interpretation and translational study design. First, it separates autophagosome-marker movement from productive flux [15,16,23]; second, it places lysosomal function and redox burden at the center of autophagy interpretation; third, it uses flavonoid-associated perturbations as methodological stress tests rather than therapeutic rankings; and fourth, it links each autophagy-associated state to candidate vulnerabilities that can be examined in clinically anchored settings such as high-risk precursor lesions, active surveillance cohorts, and pre-prostatectomy studies [7,9,10,11,12,13,14]. Tissue exposure and pharmacodynamic plausibility should then be evaluated using metabolite recovery and evidence from prostate tissue pharmacology [45,46,47,48,49,50]. By doing so, this review positions localized prostate cancer as a disease-relevant model in which autophagy, stress adaptation, organelle crosstalk, and translational relevance can be interpreted together.

### 1.1. Scope, Literature Selection Strategy, and Evidence Hierarchy

This review focuses on interpretation rather than exhaustive cataloging or quantitative effect estimation. Literature was identified through PubMed, Scopus, and Web of Science, with emphasis on studies published between 2010 and early 2026 that were most informative for localized prostate cancer, autophagic flux, lysosomal competence, flavonoid metabolism, redox signaling, and stress adaptation. Autophagy and prostate cancer background searches were anchored in prostate- and cancer-autophagy literature [2,3,8]. In contrast, flux-, lysosomal-competence-, and ferritinophagy-related searches were anchored in the autophagy-monitoring and organelle-stress literature [15,16,29,30,43]. Priority search concepts included prostate cancer, localized disease, HGPIN, active surveillance, pre-prostatectomy, flavonoids, catechins, isoflavones, autophagy, flux, lysosome, proteostasis, mitophagy, ferritinophagy, ferroptosis, and disease models.

Search and selection boundaries. The final search was completed in early 2026. Searches combined prostate cancer terms (localized prostate cancer, HGPIN, ASAP, active surveillance, pre-prostatectomy) with autophagy and organelle terms (autophagic flux, LC3, SQSTM1, lysosome, TFEB, mitophagy, ferritinophagy, ferroptosis, NRF2) and exposure terms (flavonoids, catechins, EGCG, isoflavones, genistein, daidzein, equol, apigenin, luteolin, quercetin). Studies were prioritized when they clarified disease-stage context, tissue exposure, flux-aware interpretation, lysosomal function, organelle crosstalk, or translationally interpretable endpoints. Extra-prostate studies were retained only when they informed a specific mechanistic module not yet resolved in localized prostate cancer and are interpreted here as hypothesis-generating rather than confirmatory.

Study selection was guided by the interpretive question rather than by compound identity alone. When several reports addressed the same mechanism, priority was given to studies that provided the strongest evidence tier: human tissue-linked or pathology-linked localized prostate cancer data first, broader prostate cancer data second, prostate-specific preclinical studies with flux-aware assays third, and extra-prostate mechanistic studies only when they clarified a module that remains unresolved in localized disease. Studies were not used for central interpretation when they relied only on supraphysiologic exposure, static LC3 or p62/SQSTM1 changes, or non-prostate models without a clear mechanistic bridge. In such cases, the evidence was retained only as hypothesis-generating support [17,24,38,45,46,51,52,53,54,55,56,57,58]. When evidence quality was otherwise comparable, studies with clearer disease-stage context, explicit exposure reporting, and functional flux or lysosomal assays were prioritized over purely descriptive reports.

This review was designed as a structured narrative synthesis rather than a systematic review because its main objective is interpretive rather than quantitative. No meta-analysis, formal risk-of-bias scoring, or PRISMA-style study selection flow was performed. This should be considered a limitation of scope rather than an attempt to claim systematic completeness. The field of autophagy in localized prostate cancer is heterogeneous in experimental models, exposure conditions, biomarker panels, disease windows, and endpoint definitions. Under these conditions, a pooled or strictly comparative analysis would risk treating biologically distinct readouts as equivalent. Therefore, the purpose of this review is not to estimate an overall effect size or to rank interventions, but to define conceptual and methodological criteria required to assign biological meaning to autophagy-associated marker changes.

A narrative framework is particularly appropriate here because the central problem is cross-disciplinary. Interpreting autophagy-associated readouts requires integrating autophagic flux, lysosomal competence, redox biology, mitochondrial quality control, iron handling, the realism of pharmacological exposure, and prostate cancer disease stage. The evidence was therefore organized hierarchically, prioritizing studies that link clinically relevant prostate cancer settings with tissue pharmacology, flux-aware readouts, lysosomal function, and translationally interpretable endpoints. This approach allows the review to identify recurring failure modes and propose practical standards for future studies.

Priority was given to clinically informative settings, such as HGPIN, ASAP, active surveillance, and pre-prostatectomy studies, because these designs offer the clearest opportunity to connect interventions, tissue biology, and clinically anchored short-term trajectories [7,9,10,11,12,13,14]. Human studies were considered especially informative when oral exposure could be linked to prostate tissue pharmacology, biomarker modulation, or pathology-relevant endpoints [46,47,48,49,50,54].

To make interpretation more transparent, the evidence discussed throughout this review is weighed hierarchically. The highest tier includes human intervention studies with tissue or pathology-linked endpoints. A second tier includes tissue pharmacology and metabolite recovery studies that define chemical plausibility in the prostate. A third tier includes prostate-specific preclinical models, especially those using flux-aware designs and autophagy-monitoring logic [15,16,23]. A fourth, hypothesis-generating tier includes broader mechanistic literature used only when it clarifies the meaning of autophagy-related readouts in localized prostate cancer. This extrapolated tier is particularly relevant for lysosomal competence, TFEB signaling, ferritinophagy, ferroptosis, and redox adaptation [2,3,29,30,31,32,33,42,43,59].

This hierarchy matters because many mechanistic claims become fragile once they are removed from exposure realism, lysosomal competence, or flux testing. Selected extra-prostate studies are therefore used here as interpretive scaffolds rather than substitutes for prostate-specific validation. Wherever mechanistic extrapolation is necessary, it is treated as supportive and provisional rather than confirmatory.

To avoid overinterpreting extrapolated mechanisms, evidence is classified into four interpretive levels throughout this review, as summarized in Table 1. Localized prostate cancer evidence is given the highest weight when human, tissue-linked, or pre-prostatectomy data connect exposure with pathology-relevant endpoints. Broader prostate cancer evidence supports disease-specific plausibility but is not treated as direct confirmation in localized disease. Extra-prostate mechanistic evidence is used as a mechanistic precedent only when it clarifies unresolved modules such as lysosomal competence, TFEB signaling, ferritinophagy, ferroptosis, or redox adaptation. Finally, hypothesis-generating evidence is useful for identifying future experiments but is insufficient for firm translational conclusions. These levels should be interpreted as confidence qualifiers rather than as a claim that all modules have equivalent support in localized disease.

The present review is therefore positioned against two existing literatures: reviews of autophagy in prostate cancer and of flavonoids in prostate models. Its point of difference is that it asks a narrower and more stringent question than either tradition usually does when autophagy-associated marker changes are reported in localized prostate cancer [2,3,8,15]. What minimum evidence is required before that movement can be classified as beneficial, adaptive, or artifactual?

### 1.2. Rationale for an Organelle-Centered Framework

Autophagy is not a linear marker pathway but a distributed organelle program in which autophagosomes, lysosomes, mitochondria, endosomal membranes, ferritin stores, and redox-responsive signaling nodes operate as an integrated network. This organelle-centered view is particularly relevant in localized prostate cancer, where AR signaling, PTEN/PI3K/AKT activity, mitochondrial stress, p62/SQSTM1-KEAP1-NRF2 adaptation, TFEB-linked lysosomal regulation, and peri-prostatic inflammatory cues may produce similar LC3 or p62/SQSTM1 patterns while representing different biological states. A framework focused on organelle crosstalk, therefore, offers a more appropriate basis for translational inference than directional language such as “autophagy induction” or “autophagy inhibition”.

## 2. Autophagy in Localized Prostate Cancer: Biological Duality and Disease-Stage Dependence

Autophagy in localized prostate cancer should be interpreted as stage-dependent rather than inherently beneficial or harmful. In early lesions or high-risk precursor states, autophagic turnover may limit oxidative damage, preserve organelle quality, and restrain chronic inflammatory signaling. In more stress-adapted tumor cells, however, the same machinery may stabilize metabolism, sustain survival under hypoxia or androgen stress, and protect cells from treatment-associated injury [4,18,22].

This duality is especially relevant in prostate cancer because autophagy sits at the intersection of androgen receptor signaling, PTEN/PI3K/AKT/mTOR status, nutrient sensing, mitochondrial quality control, and inflammatory tone. As a result, identical LC3 or p62/SQSTM1 patterns may correspond to very different biological states depending on the underlying signaling context and on whether the degradative arm of the pathway remains competent [4,18,19,20,21,22].

Localized disease is therefore informative, not because it guarantees a protective role for autophagy, but because it allows the field to test whether autophagy reflects restored tissue homeostasis, emerging stress tolerance, blocked clearance, or stress overload before systemic therapy and metastatic adaptation dominate the biology. This is precisely why clinically anchored endpoints, beyond PSA alone, remain important in early-disease studies, especially when linked to tissue exposure, histopathologic context, and organelle-level readouts [7,9,10,11,12,13,14].

## 3. Flavonoid-Associated Stress Perturbation Models: Recurring Interpretive Limitations

Flavonoids are useful probes precisely because they perturb multiple systems relevant to the interpretation of autophagy. Redox tone, inflammatory signaling, androgen biology, mitochondrial stress, and PI3K/AKT/mTOR signaling are supported primarily by the mechanistic literature on flavonoids and prostate cancer [24,25,26,27,28,60,61,62]. Lysosomal behavior, iron handling, ferritinophagy, and ferroptosis-linked vulnerability require separate pathway-specific interpretation [35,36,37]. Those same features also make them unusually easy to overinterpret when readouts are isolated from biological context.

The first recurring failure mode is exposure mismatch. Many in vitro studies test parent aglycones at concentrations that are difficult to reconcile with human prostate exposure, even though conjugated metabolites dominate circulating and tissue-associated flavonoid pools. Under those conditions, autophagy-related phenotypes may describe a pharmacological artifact rather than plausible tissue biology [46,47,48,49,50,63].

The second failure mode is interpretive inflation around redox markers. Reduced ROS or induction of antioxidant programs is often presented as evidence of beneficial autophagy, yet such changes may reflect adaptive buffering that preserves tumor-cell fitness. A favorable oxidative shift is not synonymous with a favorable autophagic state [19,20,25,27,28].

Representative human data illustrate the magnitude of this gap. In a randomized pre-prostatectomy tea study, 20 men consumed 1.42 L/day of green tea, black tea, or a caffeine-matched control for 5 days before radical prostatectomy; tea polyphenols were higher in prostate samples from tea-consuming groups than in controls, whereas they were not detectable in serum, underscoring that tissue recovery and plasma exposure cannot be assumed to be equivalent [54]. A later prospective randomized trial in 31 men evaluated 1 g/day green tea extract with 800 mg/day quercetin for 4 weeks before prostatectomy and explicitly quantified polyphenols in blood, urine, and prostate tissue, making it a useful exposure-realism benchmark for future flux-aware studies [45]. Soy-isoflavone supplementation provides an additional numerical anchor: median total isoflavones in supplemented men reached 2.3 micromole/L in prostate tissue versus 0.7 micromol/L in serum, with average tissue concentrations approximately six-fold higher than serum concentrations [46]. Therefore, in vitro studies using high-micromolar parent aglycones should be interpreted as pharmacological stress tests unless they are justified against measured prostate-tissue exposure and the relevant circulating or tissue-associated metabolite forms [45,46,54].

The third failure mode is treating LC3 puncta, LC3-II accumulation, or p62/SQSTM1 movement as self-interpreting endpoints. None of these markers alone can distinguish increased autophagosome biogenesis from blocked lysosomal clearance. The problem has become even sharper as the field has recognized non-canonical ATG8 conjugation to single membranes and other ATG8-positive states that are not equivalent to productive macroautophagic flux [15,16,23]. Without flux testing, the biological meaning of flavonoid-associated marker movement remains unresolved.

Where available, tandem fluorescent LC3 reporters such as mRFP-GFP-LC3 or GFP-mCherry-LC3 should be used to separate autophagosomes from acidic autolysosomes, particularly when a compound may alter lysosomal pH. In the original tandem LC3 logic, GFP and red fluorescence are both visible before lysosomal fusion. In contrast, the acid-sensitive GFP signal is lost after fusion, and the red-only signal marks autolysosomal maturation. These reporters do not replace biochemical flux blockade, LC3-II turnover, p62/SQSTM1 dynamics, or cargo-degradation assays, but they provide an orthogonal readout that helps distinguish increased LC3-positive vesicles from completed degradation [24,57,58].

A fourth problem is the routine neglect of lysosomal competence. Compounds can perturb membrane trafficking, acidification, repair pathways, or cargo turnover while still producing the appearance of ‘autophagy induction.’ In those settings, cargo accumulation may represent traffic congestion, lysosomal damage responses, or stalled degradation rather than productive recycling [29,30,31,32,33].

EGCG studies further illustrate this point, because lysosomal acidification can determine whether an ATG8/LC3-positive response represents incomplete autophagy or a more degradative state [64].

Finally, literature often collapses biologically distinct outcomes into a single label. Homeostatic turnover, adaptive tumor survival, ferritinophagy-linked stress sensitization, mitophagy-compatible quality control, and lethal stress-overload programs are not equivalent simply because they all involve autophagy-related machinery. Flavonoid studies repeatedly illustrate this conceptual slippage, especially when ferroptosis is inferred solely from changes in ROS or when ferritinophagy is invoked without NCOA4-aware evidence and rescue logic [35,36,42,43,62].

For that reason, flavonoids are treated here as mechanistic stress probes rather than as a nutraceutical ranking exercise. Their value lies in revealing where the current literature distinguishes productive autophagy from adaptive buffering, blocked clearance, or stress-overload responses, and where it still relies on unresolved marker movement. The key interpretive task is to classify the autophagy-related state created by the perturbation rather than to record a directional change.

## 4. Determinants of Autophagic Meaning: Redox Burden, Lysosomal Competence, and Disease Stage

**Figure 1:**
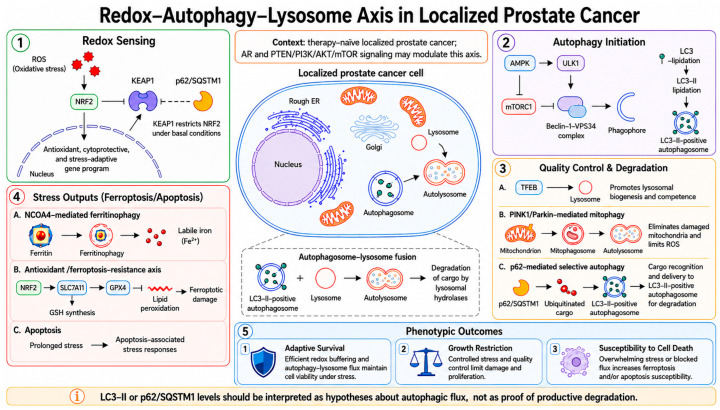
Redox-autophagy-lysosome stress-response axis in localized prostate cancer. The figure integrates redox sensing, AMPK-mTOR-ULK1 initiation, TFEB-linked lysosomal competence, p62/SQSTM1 cargo handling, mitophagy, ferritinophagy, lipid peroxidation, and phenotype-level outputs. The central message is that LC3 or p62/SQSTM1 readouts must be interpreted as hypotheses about flux rather than proof of productive degradation. Abbreviations: ROS, reactive oxygen species; NRF2, nuclear factor erythroid 2-related factor 2; p62/SQSTM1, p62/sequestosome 1; KEAP1, Kelch-like ECH-associated protein 1; AMPK, AMP-activated protein kinase; mTOR, mechanistic target of rapamycin; ULK1, Unc-51-like autophagy activating kinase 1; VPS34, vacuolar protein sorting 34; TFEB, transcription factor EB; PINK1, PTEN-induced kinase 1; GPX4/SLC7A11, glutathione peroxidase 4/solute carrier family 7 member 11; LC3, microtubule-associated protein 1 light chain 3. The numbered modules indicate: ➀ redox sensing, ➁ autophagy initiation, ➂ quality control and degradation, ➃ stress outputs, and ➄ phenotype outcome.

The meaning of autophagy in localized prostate cancer emerges from the integration of redox sensing, nutrient signaling, and degradative competence. AMPK-mTOR-ULK1 signaling, Beclin-1/VPS34-dependent nucleation, and prostate-cancer stress adaptation provide the initiation and disease-context layer [4,18,19,20,21,22,23]. p62/SQSTM1-KEAP1-NRF2 signaling, TFEB-linked lysosomal biogenesis, and lysosomal damage responses define the redox-lysosome interpretive layer [29,30,31,32,33,59]. Mitophagy, ferritinophagy, and ferroptosis-related modules add organelle-specific and iron-dependent stress controls [35,42,43]. The integrated architecture of these redox, autophagy initiation, quality control, and stress output modules is summarized in Figure 1.

To clarify why isolated marker interpretation is insufficient, Figure 1 organizes the main organelle-centered modules that shape the meaning of autophagy in localized prostate cancer, including redox sensing, AMPK-mTOR-ULK1 initiation, p62/SQSTM1 cargo handling, TFEB-linked lysosomal competence, mitophagy, ferritinophagy, lipid peroxidation, and downstream biological outputs.

The main implication of Figure 1 is that autophagy-associated markers acquire biological meaning only when they are read within this organelle network, rather than as isolated upstream or downstream signals.

Among these axes, the p62/SQSTM1-KEAP1-NRF2 interface is particularly important, as it links autophagy status to antioxidant adaptation. In some prostate-cancer settings, p62/SQSTM1 accumulation and NRF2 activation may not indicate recovery, but rather a rewired cytoprotective state that helps malignant cells tolerate chronic stress. Recent EGCG work further supports caution, as autophagy-dependent regulation of the p62/SQSTM1-mediated antioxidant survival pathway can influence selective cancer cell death. p62/SQSTM1 behavior should therefore be interpreted alongside flux, pathway context, and redox-state readouts rather than as an isolated indicator of protective autophagy [19,20,39,40,41,59].

Autophagy in prostate cancer is also closely linked to lineage signaling and lysosomal regulation. Hypoxia-associated p62/SQSTM1 phosphorylation can promote androgen receptor degradation, while lysosomal status is coupled to TFEB translocation and mTORC1 signaling, two nodes that determine whether degradative capacity is preserved or exhausted [21,22,29,30,31,32,33]. These links complicate any simple claim that changes in autophagy-related markers are inherently favorable or unfavorable. EGCG-related TFEB activation reported in renal cell carcinoma further supports the value of interpreting TFEB-linked lysosomal regulation as a mechanistic module, while still treating it as extra-prostatic evidence requiring prostate-specific validation [65].

EGCG-related studies also illustrate why lysosomal competence should be treated as an interpretive determinant rather than as a secondary assay. Zhong et al. reported that EGCG counteracted HBV-associated incomplete autophagy by improving lysosomal acidification, thereby shifting the meaning of autophagy-associated readouts from stalled or incomplete processing toward a more degradative state. For localized prostate cancer, this finding should be interpreted as a mechanistic precedent rather than direct disease-specific evidence: when flavonoids are proposed to regulate autophagy, assays of lysosomal acidification, cargo turnover, and flux blockade are necessary to determine whether the observed response reflects productive degradation or unresolved marker accumulation [63].

**Figure 2:**
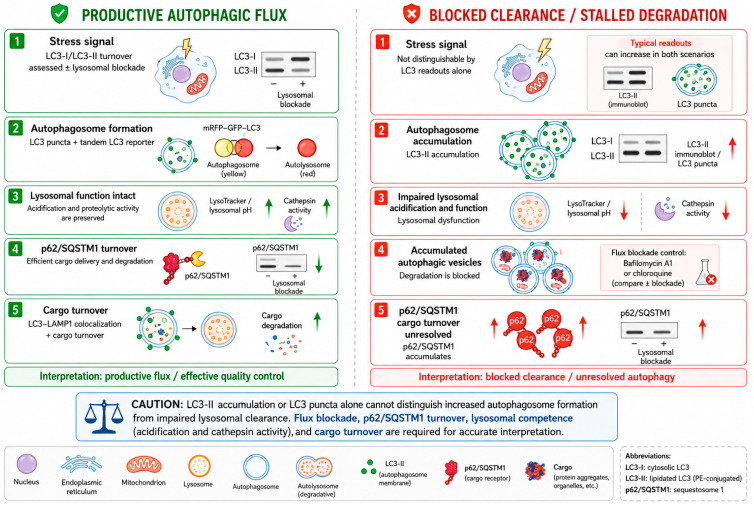
Static autophagy markers cannot distinguish productive autophagic flux from blocked clearance. The left panel summarizes productive autophagic flux, in which LC3-associated autophagosome formation is followed by autophagosome–lysosome fusion, autolysosome formation, and cargo degradation, consistent with effective quality control. The right panel summarizes blocked clearance/stalled degradation, in which LC3-positive vesicles accumulate because lysosomal acidification and degradative function are impaired, resulting in defective cargo turnover and accumulation of p62/SQSTM1. Because similar LC3-related readouts may arise from opposite biological situations, LC3-II accumulation or LC3 puncta alone are insufficient to distinguish increased autophagosome biogenesis from impaired lysosomal clearance. Commonly used experimental approaches that help resolve this distinction include LC3-I/LC3-II immunoblotting, LC3 puncta imaging, tandem fluorescent LC3 reporters, LC3–LAMP1 colocalization, p62/SQSTM1 turnover, lysosomal pH or LysoTracker-based assays, cathepsin/proteolytic activity measurements, cargo degradation assays, and pharmacologic flux blockade with bafilomycin A1 or chloroquine. Together, these assays allow flux-aware interpretation by integrating autophagosome formation, lysosomal competence, and cargo degradation.

Methodology, therefore, matters as much as biology. Static accumulation of LC3 puncta or LC3-II does not, by itself, prove increased flux. The same signal may arise from enhanced autophagosome formation, impaired lysosomal degradation, altered membrane trafficking, CASM-related ATG8 lipidation, or broader stress-associated remodeling. The same caution applies to p62/SQSTM1, whose abundance reflects both synthesis and turnover [15,16,23,59]. This distinction between productive flux and blocked clearance is illustrated in Figure 2.

Because LC3-II accumulation, LC3 puncta, and p62/SQSTM1 changes can arise from opposite biological situations, Figure 2 separates productive autophagic flux from blocked clearance. This distinction is central to the proposed framework because both states may look similar when only static markers are measured.

Thus, flux-blockade and lysosomal-competence assays are essential, not optional. Figure 2 shows that similar LC3/p62/SQSTM1 patterns may indicate either increased degradative flux or stalled clearance, making flux-blockade and lysosomal-competence assays essential rather than optional.

This is why the current autophagy-monitoring guidelines remain central to any serious interpretation. LC3 dynamics, p62/SQSTM1 behavior, pharmacological lysosomal blockades, and orthogonal imaging or colocalization approaches should be interpreted together rather than treated as interchangeable surrogates for flux [15,23].

In practical terms, flavonoid-associated autophagy claims are strongest when they show that lysosomal function remains intact, that cargo turnover is occurring, and that the resulting phenotype can be placed within a biologically coherent state rather than inferred from marker accumulation alone. Where lysosomal damage or repair is relevant, that state should also be separated from lysophagy and from endo-lysosomal damage responses more broadly [29,30,31,64,65].

From a translational standpoint, a minimum autophagy package should therefore include at least one marker of autophagosome formation, one marker of cargo turnover, and one assay that directly addresses lysosomal competence or pharmacological flux blockade. In ferritinophagy-linked settings, NCOA4-aware readouts and ferroptosis-specific controls are also needed; in mitophagy-linked settings, mitochondrial quality-control measurements should be explicit rather than assumed [42,43]. These minimum criteria are summarized in Table 2.

Not every pathway discussed in this review has been established directly in prostate-specific systems. Some components, particularly ferroptosis-linked responses and certain forms of flavonoid-induced redox stress, are informed in part by broader cancer literature. Those parallels are useful for interpretation, but they should not be treated as equivalent to prostate-specific confirmation [35,36,42,43,62].

Accordingly, the mechanistic maps and case studies below apply this evidence-status logic to distinguish prostate-specific support from mechanisms that are extrapolated or hypothesis-generating. The tables should therefore be read as interpretive tools, not as claims that every pathway has equivalent validation in localized prostate cancer. The minimum evidentiary standard required to assign autophagic meaning in localized prostate cancer is summarized in Table 3. This standard emphasizes that LC3-II accumulation, LC3 puncta, p62/SQSTM1 abundance, lysosomal status, and phenotype-level outcomes cannot be interpreted in isolation. Instead, each readout must be paired with complementary assays that clarify flux, lysosomal competence, cargo turnover, pathway specificity, and disease-stage relevance before any biological or translational meaning is assigned.

Operationalizing the decision framework requires a strict stepwise approach: from observed readout to required control, and finally to a provisional biological state. This sequence ensures that mere shifts in static markers are not mistakenly interpreted as therapeutic benefits. The stepwise decision logic is summarized in Table 4.

The proposed framework also requires pathway-specific interpretation, as distinct stress-response modules may yield overlapping autophagy-associated readouts. Therefore, redox signaling, p62/SQSTM1-KEAP1-NRF2 adaptation, AMPK-mTOR-ULK1 initiation, PI3K/AKT/mTOR survival signaling, inflammatory stress, TFEB-linked lysosomal regulation, ferritinophagy, ferroptosis, and mitophagy should not be collapsed into a single directional label of autophagy induction or inhibition. The main pathway modules, core nodes, recommended readouts, and interpretive caveats are summarized in Table 5.

## 5. A Flux-Aware Framework for Interpreting Autophagy Responses

The decision framework is applied through three linked questions: which disease window is being modeled, what level and type of redox stress is being imposed, and whether the lysosomal compartment remains sufficiently competent to complete cargo degradation. These questions convert isolated marker changes into a provisional biological state rather than a directional claim.

**Figure 3:**
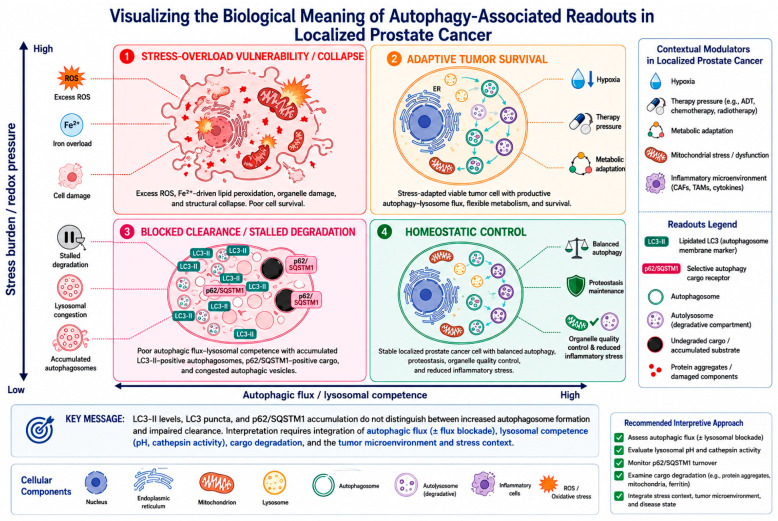
Four biologically distinct autophagy-associated stress-response states in localized prostate cancer. Stress burden/redox pressure and autophagic flux/lysosomal competence jointly classify autophagy-associated readouts as homeostatic control, adaptive tumor survival, blocked clearance, or stress-overload vulnerability. These categories are interpretive states rather than fixed disease entities.

Disease stage defines the biological window, redox burden determines whether stress is buffered or amplified, and lysosomal competence determines whether the signal represents productive degradation or congestion within the degradative system. Together, stress burden/redox pressure and lysosomal competence generate the four-state model shown in Figure 3.

The four-state model is not intended to replace established autophagy-monitoring guidelines. Rather, it translates flux-monitoring principles and lysosome-centric interpretation into disease-relevant categories for localized prostate cancer. In this respect, the model extends previous flux-based and prostate cancer autophagy frameworks by adding two contextual axes that are often underdeveloped in prostate cancer reviews: realistic tissue exposure and disease-window-specific biological meaning [15,16,17,24,38,57,58].

This model is intended as a classification tool, not as a claim that all four states have equivalent levels of direct evidence in localized prostate cancer. Operationally, each state requires a different combination of flux-aware assays, lysosomal-competence readouts, pathway-specific controls, and phenotype-level endpoints. The evidence requirements, biological interpretation, and candidate translational vulnerabilities associated with each state are summarized in Table 6.

This model is intended as a classification tool, not as a claim that all four states have equivalent levels of direct evidence in localized prostate cancer.

Operationally, homeostatic control is assigned when intact flux and lysosomal competence reduce damage without preserving malignant fitness; for example, low redox burden with cargo clearance in early lesions or surveillance tissue would fit this state. Adaptive tumor survival is conferred when the same intact flux supports viable tumor cells under hypoxia, androgen stress, therapeutic pressure, or metabolic strain. Thus, the boundary between the two states is not the presence of flux itself, but the phenotype and disease context coupled to that flux.

Redox burden matters because flavonoids are not fixed antioxidants. At modest stress intensity, they may dampen inflammatory or mitochondrial stress enough to support quality control and proteostasis. At higher intensity, or in cells already near the threshold for oxidative or iron-dependent injury, the same compounds may amplify vulnerability and drive the system toward apoptosis- or ferroptosis-linked collapse [24,25,26,27,28,35,36]. This context dependence is also illustrated by EGCG studies in melanoma, NSCLC, and hepatoma models, where EGCG either decreased autophagy or leveraged autophagy inhibition to enhance drug-induced cell death, reinforcing the notion that autophagy modulation cannot be assigned a single direction across systems [66,67,68].

Lysosomal competence is the third and often neglected axis. If degradation remains intact, increased autophagosome formation may indicate productive flux. If lysosomal function is compromised, however, similar marker changes may signify congestion within the degradative system. Without addressing this distinction, translational claims remain unstable [4,18,22,23].

A flavonoid-associated autophagy claim should be considered interpretable only when realistic exposure is linked to a defined readout package: markers of autophagosome formation, cargo turnover, lysosomal competence, and a phenotype consistent with homeostatic control, adaptive tumor survival, blocked clearance, or stress-overload vulnerability. At that level, flavonoid-associated biology is well-suited to mechanistic and translational inference [15,16,23,64,69].

## 6. Stress-Test Case Studies for Organelle-Centered Autophagy Interpretation

The flavonoids most often discussed in localized prostate cancer should not be treated as a uniform therapeutic class. They are more informative when read as distinct stress perturbation case studies that expose where autophagy interpretation is relatively strong, where it remains chemically implausible, and where marker inflation continues to distort conclusions. These case studies are not intended to rank flavonoids therapeutically, but to test how the proposed framework handles different levels of exposure realism, mechanistic support, flux resolution, lysosomal competence, and disease relevance.

To avoid treating all compounds at the same evidence level, the case studies are stratified by the strength of human evidence, tissue pharmacology, and flux-aware validation, with human prostate tissue studies as the highest-exposure anchor and autophagy-monitoring guidance as the methodological anchor [24,45,46,54,57,58]. EGCG/green-tea catechins and soy isoflavones provide the most useful human or tissue-linked anchors, while quercetin, apigenin, luteolin, and carnosic acid are preclinical or mechanistic probes. Curcumin and resveratrol are included as non-flavonoid polyphenolic comparators to demonstrate that the framework is not limited to flavonoids [52,55,56]. This evidence-stratification logic is summarized in Table 7.

The representative flavonoid-associated perturbation case studies used to stress-test the framework are summarized in Table 8. These examples are not intended to rank compounds therapeutically, but to show how different levels of exposure realism, redox modulation, lysosomal competence, pathway specificity, and disease relevance influence the interpretation of autophagy-associated readouts in localized prostate cancer.

### 6.1. When Tissue Exposure and Autophagy Readouts Begin to Converge

Green tea catechins provide one of the clearest examples of how tissue exposure and autophagy interpretation can be examined within the same disease window in localized prostate cancer, because they have been studied in HGPIN cohorts, pre-prostatectomy settings, and tissue-recovery designs in humans [9,10,11,12,46,47,48]. This does not establish benefit on its own, but it makes catechins a useful interpretive benchmark for assessing whether exposure, sampling, and autophagy readouts are aligned.

Their value for the present review lies in the distinction between interpretable and weakly interpretable evidence. Stronger catechin studies link oral intervention to tissue recovery, controlled exposure, or biologically anchored sampling [9,10,11,12,46,47,48]. By contrast, weaker mechanistic claims invoke high-dose in vitro LC3-II accumulation, EGCG-associated survival effects, or generic antioxidant responses without resolving metabolite realism, flux, or lysosomal function [33,34,40,64,70,71,72,73].

Pre-prostatectomy trials are particularly valuable because they do not need to establish long-term effectiveness to provide useful information. Their primary benefit is that they link drug intake, exposure to the target organ, and pharmacodynamic measurements within the same patient. This connection is exactly what the study of autophagy has been lacking in much of the field [12].

Modernelli et al. provides a prostate-specific example of why EGCG cannot be treated as a simple anticancer autophagy inducer. In prostate cancer cells, EGCG antagonized bortezomib cytotoxicity via an autophagic mechanism, suggesting that EGCG-associated autophagy may support survival in some therapeutic contexts rather than uniformly promote tumor suppression. This finding is especially useful for the present framework because it links EGCG, prostate cancer, therapeutic stress, and context-dependent autophagy within a single experimental system [31].

Beyond prostate-specific evidence, EGCG studies in other systems reinforce the need to resolve the degradative arm of the pathway. Zhong et al. showed that EGCG can oppose incomplete autophagy by enhancing lysosomal acidification, whereas Kim et al. reported that EGCG-induced autophagic flux can reduce TRAIL-induced apoptosis. These examples support the central point of this review: EGCG-related signals should be classified by flux, lysosomal competence, and phenotype rather than by LC3 or p62/SQSTM1 movement alone [63,68].

Catechins, therefore, serve as a benchmark for what an autophagy-oriented study looks like when exposure, tissue sampling, flux resolution, lysosomal readouts, and phenotype-level interpretation are aligned.

### 6.2. Soy Isoflavones, Daidzein, and Equol: Interpretation Requires Metabolic Stratification

Soy isoflavones remain compelling because they sit at the intersection of redox biology, endocrine signaling, microbiome-dependent metabolism, and tissue pharmacology. In localized prostate cancer, that combination makes them highly relevant but also unusually heterogeneous [7,13,54].

The strongest interpretation arises when short-term intervention is linked to measurable exposure and to pathway-aware biological sampling, as in pre-prostatectomy work with genistein [13]. The weakest interpretation appears when all participants are treated as chemically equivalent despite major differences in microbial conversion and metabolite production.

Equol status is especially important in this respect. Because only a subset of individuals efficiently converts daidzein to equol, similar interventions may generate substantially different exposures among patients. Ignoring that variability can blur real biological differences and make autophagy-related readouts appear inconsistent when the underlying chemistry is heterogeneous [54,74,75].

Accordingly, soy interventions are most informative when metabolite identity, equol-producer phenotype, and tissue-level exposure are built into the design. Without that stratification, the interpretation of autophagy remains chemically underdetermined. This point expands the equol-producer phenotype into a broader host-metabolic stratification problem. Microbiome-dependent biotransformation can change the chemical species that reach the prostate, while obesity, diet, antibiotic exposure, and inflammatory tone may reshape redox state and lysosomal fitness. Future isoflavone studies should therefore predefine equol-producer status and, when feasible, profile microbial metabolites rather than treating all participants as chemically equivalent.

### 6.3. Apigenin and Luteolin: Mechanistic Probes of Stress-Program Interpretation

Apigenin and luteolin are often discussed together as broad anticancer flavones, but they are more useful as mechanistic probes than as independent translational candidates. Their main value lies in the autophagy-related questions they help clarify, rather than in any straightforward ranking of clinical potential [36,76].

Luteolin is particularly useful because it brings ferritinophagy, TFEB signaling, iron handling, and ferroptotic vulnerability more directly into the discussion than most other compounds in this review. That makes it a powerful example of why ROS alone is insufficient; iron flux, lipid peroxidation, GPX4/SLC7A11 status, and rescue controls are necessary to classify the induced state [35,36,62]. A recent luteolin-focused review now provides background on the broader pharmacological context of luteolin. At the same time, the ferroptosis/ferritinophagy claim remains anchored in prostate-specific mechanistic evidence where available [53].

Therefore, ferritinophagy- and ferroptosis-related modules are presented here as interpretive extensions that require prostate-specific validation, rather than as established mechanisms in localized prostate cancer.

Apigenin, by contrast, is frequently discussed in terms of apoptosis or autophagy-associated markers without equivalent attention to pharmacological realism or flux resolution. It therefore illustrates how quickly mechanistic richness can outpace interpretive discipline when autophagy is inferred rather than demonstrated [8,34,66].

Taken together, these flavones are best seen as tools for stress-program interpretation: they can clarify when lysosomal engagement, ferritin turnover, or redox amplification is being mistaken for a generic anticancer autophagy signal.

### 6.4. Quercetin and Related Flavonols: The Problem of Static Marker Inflation

Quercetin illustrates one of the field’s most persistent interpretive problems: the tendency to convert any shift in LC3- or p62/SQSTM1-associated readouts into evidence of favorable autophagy. Because quercetin is widely available and mechanistically versatile, it is often used to support broad claims that exceed the actual strength of the data [26,27,28].

That does not make quercetin unimportant. On the contrary, it may be one of the more useful system-level modulators in the group. The problem is that its literature is especially prone to static-marker inflation, exposure mismatch, and overgeneralized antioxidant language. In that sense, quercetin is a case study in how a plausible compound can still be interpreted poorly.

Human data remain informative mainly for feasibility and tolerability, not for strong claims about autophagy-driven efficacy. Quercetin is therefore more useful here as a cautionary example of interpretive excess than as a compound to be advanced based on static-marker changes alone.

Human studies that help classify, rather than merely claim, autophagy-associated responses in localized prostate cancer are summarized in Table 9. These studies are most useful when they connect oral exposure, prostate tissue recovery, metabolite identity, pathology-linked endpoints, and biologically interpretable readouts. However, even in these clinically anchored settings, autophagy-related conclusions remain provisional unless tissue exposure is paired with flux-aware assays, lysosomal-competence measurements, and phenotype-level interpretation.

Human studies remain valuable in this field, but their value is interpretive rather than automatically confirmatory. They help define which classes have realistic exposure, which trial designs permit tissue sampling, and which biomarkers can be tied to clinically meaningful windows of disease [7,9,10,11,12,13,14].

Taken together, the human studies literature should be synthesized by clinical window rather than by compound enthusiasm. HGPIN studies are useful for prevention-oriented biological signals; active-surveillance cohorts are useful for longitudinal risk stratification; and pre-prostatectomy window studies are the strongest design for pairing exposure with tissue pharmacodynamics and organelle-level readouts. The key limitation is that most human studies do not yet include a complete autophagy flux and lysosomal competence package.

The Pomi-T trial illustrates both the promise and the interpretive limit of complex polyphenol-rich interventions. Such mixtures may generate measurable biological signals, but once several bioactive components are combined, it becomes much harder to attribute an autophagy-related state to any one compound or mechanism [14].

For that reason, the present evidence base supports neither broad dismissal nor generic optimism. Flavonoids remain useful only insofar as they help classify what kind of autophagy-related biology is produced under realistic conditions. Progress depends on clearly defined interpretive criteria rather than enthusiasm for any compound class.

The most informative evidence still comes from pre-prostatectomy studies and well-defined high-risk cohorts, where exposure can be linked to tissue pharmacodynamics. Studies focused mainly on PSA modulation or on heterogeneous mixtures are comparatively weaker for interpreting autophagy, even when they are biologically suggestive [7,9,10,11,12,13,14].

## 7. Minimum Standards for Interpreting Autophagy-Associated Stress Responses in Localized Prostate Cancer

First, the chemical species under study should be pharmacologically plausible. For flavonoid-related stress perturbations, the parent aglycone tested in vitro should not be treated as a direct substitute for the conjugated or metabolized species recovered in plasma or prostate tissue. For non-dietary perturbations, the same principle applies: dose, duration, cell state, and tissue accessibility should be compatible with the disease window being modeled.

Second, autophagy must be assessed through flux-aware logic. LC3 and p62/SQSTM1 should be interpreted together, with lysosomal blockades or complementary assays to determine whether cargo turnover is occurring. Reviewers should be able to tell from the methods whether a reported state reflects productive flux, CASM-related ATG8 lipidation, stalled degradation, or altered lysosomal acidification [15,16,23,64,69].

Constructive benchmarks are available. Stronger experimental designs do not merely report LC3-II or p62/SQSTM1; they pair these markers with lysosomal blockade, tandem LC3 reporters, lysosomal acidification, cargo turnover, viability, or rescue logic and pathway-specific markers such as NCOA4 or GPX4/SLC7A11 when ferritinophagy or ferroptosis is invoked. These examples convert a critique of static markers into a practical experimental standard.

Third, tissue and host context should be made visible. The equol-producing phenotype and microbial metabolite heterogeneity should be treated as exposure modifiers [44,74,75]. Obesity, peri-prostatic adipose signaling, inflammatory tone, AR status, PTEN/PI3K/AKT context, and oxidative baseline can further reshape the meaning of the flavonoid-associated autophagy signal [19,20,27,28,35,39,60,61,62]. Lysosomal competence should be treated as a distinct determinant rather than subsumed under generic stress biology [15,16,23,29,30,31,32,33]. Fourth, mechanistic movement should be paired with clinically interpretable anchors. Tissue pharmacodynamics, pathology-linked endpoints, repeat biopsy, imaging progression, or clearly staged surveillance metrics are more informative than biomarker drift in isolation [7,9,10,11,12,13,14].

### Common Failure Modes and Corrective Standards

A recurring failure mode is exposure mismatch: the biological conclusions are based on conditions unlikely to occur in the human prostate. The corrective standard is to anchor in vitro work to tissue-relevant concentrations or to explicitly justify departures from them.

A second failure mode is static-marker inflation: LC3-II accumulation, puncta formation, or changes in p62/SQSTM1 are reported as though they resolved flux on their own. The corrective standard is a minimal assay package that directly assesses cargo turnover and lysosomal competence and, where relevant, excludes CASM-related misclassification [15,16,23].

A third failure mode is analytical distortion introduced during tissue handling. Ex vivo deconjugation can exaggerate apparent aglycone recovery, thereby inflating claims about which chemical species were truly present in vivo. The corrective standard is rigorous reporting of sample-processing and metabolite-recovery conditions [49,50].

A fourth failure mode is unmodeled biological heterogeneity. Equol status and microbial-metabolite production can alter exposure in ways that should be anticipated analytically [54,74,75]. Inflammatory tone, obesity-associated adipose signaling, and tumor metabolic context can modify the interpretation of stress responses [60,61,62], whereas baseline lysosomal fitness can shift the same marker pattern toward productive flux or blocked clearance [29,30,31,32,33]. The corrective standard is a stratified design rather than a retrospective explanation.

Taken together, the main barriers are exposure mismatch, analytical distortion, static-marker inflation, unmodeled biological heterogeneity, and premature claims that conflate ferritinophagy- or mitophagy-associated signatures with resolved autophagic flux.

## 8. Study-Design Priorities for Organelle-Centered, Flux-Aware Autophagy Studies

Future studies should remain anchored in clinical settings where autophagy interpretation can be tested, including active surveillance cohorts, HGPIN or ASAP-associated studies, pre-prostatectomy intervention windows, and tissue-linked pharmacodynamic designs. In these settings, autophagy-associated readouts should be interpreted only when exposure realism, disease-stage context, flux resolution, lysosomal competence, and phenotype-level endpoints are evaluated together. The main interpretive barriers and corresponding design corrections are summarized in Table 10.

Future studies should remain anchored in clinical settings where autophagy interpretation can actually be tested, particularly HGPIN, ASAP, active surveillance, and pre-prostatectomy cohorts. These settings allow the study of tissue biology, pharmacodynamic sampling, and short-term disease behavior together before late-stage selection pressures dominate the phenotype.

Pharmacology must move from a supporting role to a central one. Plasma measurements alone are insufficient; what matters is whether flavonoid-derived species reach the prostate, in what chemical forms they arrive, and whether those species engage the pathways later invoked to explain the phenotype.

The same principle applies to autophagy. Studies that rely solely on LC3-II accumulation do not tell us whether a compound promotes productive flux, stalls lysosomal clearance, corrects incomplete degradation, or triggers a broader stress program that happens to alter autophagy-related markers. Trial designs should therefore treat flux resolution and lysosomal function as requirements rather than optional mechanistic add-ons [15,16,23,64,69]. Examples in EGCG-treated systems show that this package should include the degradative phase itself, because lysosomal acidification, lysosomal membrane integrity, and autophagic flux can change the biological interpretation of the same marker pattern [17,64,69].

The host context deserves equal attention. Obesity, peri-prostatic adipose tissue, inflammatory tone, and tumor molecular background are not secondary variables; they can reshape redox stress, lipid handling, and the very meaning of autophagy-related readouts.

**Figure 4:**
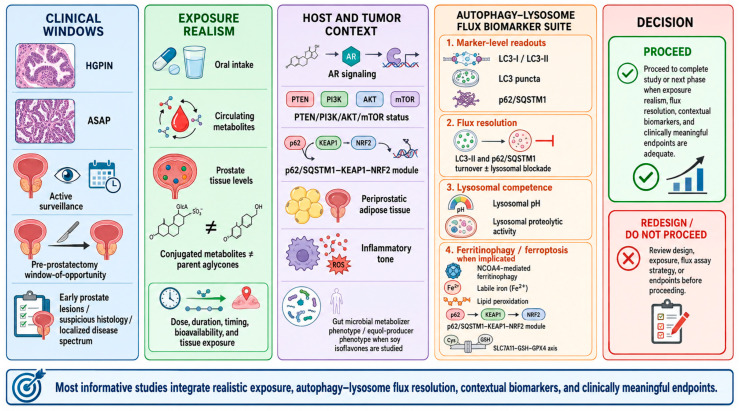
Recommended decision workflow for advancing autophagy-associated findings from mechanistic observation to disease-relevant interpretation. Progression should depend on convergence among realistic exposure, flux resolution, organelle function, and phenotype-level endpoints. Discordant signals should trigger study redesign rather than stronger mechanistic claims.

A stronger next-generation trial should therefore predefine a biomarker package aligned with the mechanism: circulating metabolite profiling, tissue compound quantification when feasible, LC3 plus p62/SQSTM1 plus lysosomal pH or proteolytic-activity assays, cargo-turnover measurements, and pathway-specific modules such as p62/SQSTM1-NRF2 signaling or iron and lipid peroxidation markers when ferritinophagy or ferroptosis is implicated [40,41,44,64,69]. A study-design workflow based on this biomarker package is shown in Figure 4.

For example, a pre-prostatectomy study could combine circulating metabolite profiling, quantification of prostate tissue compounds, LC3 and p62/SQSTM1 dynamics with lysosomal blockade or a tandem LC3 reporter, lysosomal pH or cathepsin activity, cargo-degradation assays, and paired tissue endpoints such as proliferation, oxidative damage, inflammatory tone, or pathology-linked features. Such a design would not simply ask whether autophagy increased; it would ask which autophagy-associated state was produced and whether that state created a testable organelle-centered vulnerability.

Figure 4 translates the conceptual framework into study-design priorities. It outlines how future studies should connect clinically relevant disease windows, realistic exposure conditions, host and tumor contexts, flux-aware biomarker packages, and decision points to advance or redesign translational studies.

Study advancement should depend on whether exposure, flux, organelle function, and phenotype point to the same biological state; discordant signals should trigger redesign rather than stronger mechanistic claims.

Future studies should also integrate prostate-specific metabolic rewiring, particularly lipid metabolism and androgen-linked bioenergetics, because these factors influence whether redox modulation is buffered, exploited, or pushed toward lethal stress [60,61,62].

Different perturbations, including flavonoids, are best understood by testing them across localized disease rather than as entries in a single hierarchy of promise. In some study designs, the main question is tissue exposure; in others, it is metabolic stratification, lysosomal competence, ferritinophagy-aware classification, or the separation of adaptive buffering from stress overload.

Non-flavonoid examples illustrate the same logic. Carnosic acid, curcumin, and resveratrol can all generate autophagy-associated or organelle-stress readouts. However, those readouts should still be classified by exposure realism, flux, lysosomal competence, and phenotype rather than by the compound class itself [52,55,56]. The inclusion of these examples, therefore, strengthens rather than broadens away from the central argument: prostate-specific or compound-specific activity is insufficient unless the observed LC3/p62/SQSTM1 pattern is resolved with flux and degradative controls [24,38,57,58]. This makes the framework a general interpretive tool for stress-response biology, not a flavonoid-specific argument.

In localized prostate cancer, flavonoid responses are used here as examples of a broader problem: autophagy-related claims become interpretable only when readouts are linked to exposure realism, flux, degradative capacity, and disease-stage context. Their value in this review lies as much in methodological as in biological grounds, because they serve as demanding test cases for determining whether observed readouts support restored homeostasis, adaptive tumor survival, blocked clearance, or stress-overload vulnerability.

The most credible subset of literature comprises studies that link realistic exposure to tissue pharmacology and biomarker packages capable of resolving the meaning of autophagy. This is where the field can move from descriptive claims to testable interpretive criteria.

### 8.1. Why This Organelle-Centered Framework Matters

Autophagy remains one of the most frequently invoked but often insufficiently resolved mechanisms in prostate cancer biology. In localized disease, this problem is especially important because early lesions, active surveillance cohorts, and pre-prostatectomy windows offer a unique opportunity to determine whether autophagy reflects tissue-level quality control, tumor-cell adaptation, blocked degradation, or a therapeutically exploitable stress state. If autophagy-associated readouts are interpreted without accounting for flux, lysosomal competence, exposure realism, and disease-stage context, the field risks overinterpreting marker changes as mechanistic evidence.

The practical contribution of this review is therefore not the proposal of a single compound class, biomarker, or therapeutic direction. Its main value lies in establishing an interpretive framework that helps distinguish biologically meaningful autophagy from static-marker inflation. By integrating lysosomal function, redox burden, p62/SQSTM1-NRF2 signaling, mitophagy, ferritinophagy, ferroptosis-aware controls, and clinically anchored prostate cancer settings, this review provides a more disciplined basis for deciding whether an autophagy-associated response is homeostatic, adaptive, blocked, or vulnerability-generating.

This distinction has direct translational relevance. A treatment or exposure that increases LC3-II or alters p62/SQSTM1 cannot be assumed to promote beneficial autophagy. It may instead preserve tumor-cell fitness, reflect defective lysosomal clearance, or indicate that cells are approaching a threshold of organelle stress that could be exploited therapeutically. For this reason, future studies should move beyond the language of autophagy induction or inhibition and toward state-based interpretation grounded in flux, lysosomal competence, and phenotype-level consequence.

In this sense, localized prostate cancer should be viewed not only as a disease context but also as an experimental window in which autophagy biology can be interpreted with greater clinical precision. The framework proposed here may help investigators design stronger mechanistic studies, reviewers evaluate autophagy-related claims more consistently, and translational researchers prioritize candidate organelle-centered vulnerabilities for future testing that are otherwise obscured by static readouts.

### 8.2. Limitations of the Framework

This review has limitations that should be acknowledged explicitly before translational conclusions are drawn. First, it is a structured narrative synthesis rather than a systematic review and therefore does not provide pooled effect estimates or formal risk-of-bias scoring. Second, several mechanistic modules discussed here, particularly TFEB-linked lysosomal regulation, ferritinophagy, ferroptosis-aware stress outputs, and selected flavonoid-autophagy interactions, rely partly on broader prostate cancer or extra-prostate evidence. Third, most available studies do not simultaneously measure realistic exposure, autophagic flux, lysosomal competence, cargo turnover, and phenotype-level endpoints. The proposed framework should therefore be used as a decision tool for assigning provisional biological meaning, not as definitive proof that any compound, pathway, or state has been fully validated in localized prostate cancer. Fourth, the framework is deliberately conservative: mechanisms derived from extra-prostate systems, mathematical modeling, or broader cancer biology are used to define testable hypotheses rather than to substitute for direct localized prostate cancer validation [51].

### 8.3. Broader Applicability to Other Solid Tumors

Although developed for localized prostate cancer, the framework is transferable to other solid tumors in which stress biology, lysosomal function, and tissue pharmacology remain unresolved. Its direct transfer should be approached with caution: each tumor type will require its own disease-window anchors, exposure measurements, and organelle-specific readouts. The general principle, however, is broadly applicable: autophagy-associated marker movement becomes biologically informative only when flux, lysosomal competence, redox burden, tissue exposure, and phenotype converge.

## 9. Conclusions

Overall, this review argues that autophagy in localized prostate cancer should be interpreted as a state-dependent stress-response program and organelle-centered process rather than as a directional marker of benefit or harm. The central translational task is not to determine whether autophagy is increased or decreased, but to define whether the observed response represents productive flux, adaptive tumor survival, blocked lysosomal clearance, or stress-overload vulnerability. A flux-aware and organelle-centered approach can improve the biological interpretation of existing evidence, strengthen future study designs, and help prioritize candidate vulnerabilities that emerge from crosstalk among lysosomal, mitochondrial, redox, proteostatic, and iron-handling processes. This framework is particularly relevant for localized prostate cancer, where clinically accessible disease windows still allow simultaneous study of tissue exposure, organelle function, and early translational endpoints. Future studies should therefore move from marker-based reporting of autophagy to state-based interpretation grounded in flux, lysosomal competence, realistic exposure, and disease-stage context.

## Figures and Tables

**Table 1:** The evidence-status matrix is used to distinguish direct evidence of localized prostate cancer from extrapolated or hypothesis-generating evidence.

Evidence Tier	What Counts in This Review	How It Is Interpreted	Typical Use in the Framework
Direct localized prostate cancer evidence	Human, tissue-linked, pre-prostatectomy, active surveillance, HGPIN/ASAP, or pathology-linked studies.	Highest confidence for disease-relevant interpretation.	Anchors exposure realism, clinical windows, and translational claims.
Broader prostate cancer evidence	Cell line, organoid, xenograft, advanced disease, or prostate-specific mechanistic studies [24,57,58].	Supports prostate cancer plausibility, but is not treated as direct localized disease confirmation.	Helps define AR/PTEN/PI3K/AKT/mTOR, redox, survival, and therapy-stress context.
Extra-prostate mechanistic evidence	Studies in other tumors or disease models addressing lysosomal acidification, TFEB, NCOA4, ferritinophagy, or ferroptosis.	Mechanistic precedent only requires prostate-specific validation.	Used to interpret unresolved organelle-stress modules without overstating certainty.
Hypothesis-generating evidence	Models, mathematical frameworks, compound analogies, or non-prostate observations without localized disease validation.	Useful for experimental design, not for firm translational conclusions.	Identifies future tests and clarifies the limits of the current evidence [51].

**Table 2:** Checklist for flux-aware interpretation of autophagy-associated readouts in localized prostate cancer.

Decision Step	Minimum Assay or Evidence	What It Resolves	Design Implication
Autophagosome-marker movement	LC3-I/LC3-II immunoblotting, LC3 puncta and p62/SQSTM1 dynamics.	Defines the initial observation but does not establish flux.	Treat marker movement as a hypothesis, not a conclusion. Recommended reporter and flux-assay guidance is supported by tandem LC3 and autophagy-monitoring approaches [24,57,58].
Flux resolution	Bafilomycin A1 or chloroquine blockade, tandem LC3 reporter, cargo-turnover assay.	Distinguishes increased formation from impaired degradation.	Do not claim productive autophagy without a degradative readout.
Lysosomal competence	Lysosomal pH, cathepsin/proteolytic activity, and LC3-lysosome colocalization.	Separates intact degradation from stalled or incomplete clearance.	Classify blocked clearance separately from adaptive flux.
Pathway specificity	NCOA4, labile iron, lipid peroxidation, GPX4/SLC7A11, mitophagy markers, and rescue logic.	Prevents overcalling ferritinophagy, ferroptosis, or mitophagy from ROS alone.	Use pathway-specific controls before assigning stress-overload vulnerability.
Clinical/exposure anchor	Disease window, tissue concentrations, metabolite identity, AR/PTEN context, and phenotype-level endpoint.	Determines whether the mechanism is plausible in localized prostate cancer.	Advance only when exposure, flux, organelle function, and phenotype converge.

**Table 3:** Minimum evidentiary standard for assigning autophagic meaning in localized prostate cancer.

Readout	What It Mean	What It Cannot Prove Alone	Required Complementary Assay	Main Interpretive Risk
LC3-II or LC3 puncta	Autophagosome formation, blocked degradation, CASM-related ATG8 lipidation, or stress-associated membrane remodeling.	Productive flux, increased degradation, or biological benefit.	Lysosomal blockade, tandem LC3 reporter, colocalization with lysosomal markers, and cargo-turnover assay.	Treating accumulation as proof of productive autophagy.
p62/SQSTM1 abundance	Cargo-receptor turnover, transcriptional induction, KEAP1/NRF2-linked adaptation, or defective clearance.	Protective autophagy or cargo turnover without flux-aware testing.	Flux-aware p62/SQSTM1 dynamics, NRF2 target expression, and paired viability or stress assays.	Interpreting p62/SQSTM1 accumulation as protective autophagy without testing turnover.
Lysosomal status	Preserved degradation, impaired acidification, lysosomal overload, damage response, or lysophagy-related remodeling.	Completion of autophagic degradation without cargo and functional readouts.	Lysosomal pH, proteolytic activity, cathepsin function, cargo degradation, and pharmacologic flux blockade.	Missing, stalled, or incomplete autophagy due to impaired degradative capacity.
Phenotype-level outcome	Homeostatic control, adaptive tumor survival, blocked clearance, or stress-overload vulnerability.	Clinical relevance, causality, or stage-specific benefit without matching endpoints and rescue logic.	Stage-appropriate endpoints, realistic exposure, tissue pharmacodynamics, and pathway-specific rescue logic.	Confusing mechanistic plausibility with translational relevance.

**Table 4:** Stepwise interpretive safeguards for assigning autophagy-associated biological states.

Step	Key Question	Minimum Evidence to Check	Interpretive Safeguard
1. Marker movement	Which readout changed?	LC3-II, LC3 puncta, p62/SQSTM1, cargo markers, and imaging context.	Treat the readout as a hypothesis about flux, not as proof of degradation.
2. Degradative arm	Is lysosomal function preserved?	Lysosomal blockade, lysosomal pH, cathepsin/proteolytic activity, and cargo turnover.	Separate productive flux from blocked clearance or incomplete degradation.
3. Biological context	What disease and exposure window is being modeled?	Localized disease stage, realistic exposure, AR/PTEN/PI3K/AKT context, redox baseline, and host factors.	Avoid generalizing extra-prostate or supraphysiologic findings as direct evidence of localized disease.
4. State assignment	Which biological state is most consistent?	Phenotype-level endpoints, rescue logic, and pathway-specific controls.	Classify the response as homeostatic control, adaptive tumor survival, blocked clearance, or stress-overload vulnerability only when evidence converges.

**Table 5:** Mechanistic map of the pathways and minimum interpretive readouts required to classify autophagy-related states in localized prostate cancer.

Pathway Module	Core Nodes	Relevance to Localized Prostate Cancer	Representative Flavonoids	Expected Biological Consequence	Minimum Recommended Readouts	Main Interpretive Caveat	Representative References
KEAP1/NRF2/ARE antioxidant signaling	KEAP1, NRF2, antioxidant response element, and downstream cytoprotective genes.	Supports adaptation to oxidative stress and may favor survival under chronic metabolic or inflammatory pressure.	Catechins, isoflavones, quercetin, flavonoid-rich mixtures.	Cytoprotecting at modest stress levels; possible resistance-supporting adaptation if persistently activated.	NRF2 nuclear localization, downstream target expression, oxidative stress markers, and paired viability/stress assays.	NRF2 activation does not automatically imply tumor suppression; in some contexts, it may support malignant fitness.	[19,20,22,26,28,39,40]
p62/SQSTM1-KEAP1-NRF2 interface	p62/SQSTM1, KEAP1 sequestration, NRF2 activation.	Integrates autophagy status with antioxidant signaling and may link defective clearance to tumor-promoting adaptation.	Mechanistically relevant across multiple classes, especially where autophagy-related signaling is claimed.	Reinforcement of antioxidant programs under impaired or rewired proteostatic conditions.	p62/SQSTM1 protein dynamics, KEAP1 interaction, NRF2 activation, flux-aware autophagy assays.	p62/SQSTM1 accumulation may reflect impaired degradation rather than productive autophagy.	[19,20,22,24,39,40]
AMPK-mTOR-ULK1 autophagy initiation axis	AMPK, mTOR, ULK1, Beclin-1/VPS34.	Connects nutrient sensing and stress adaptation to autophagosome initiation.	Catechins, quercetin, isoflavone-related compounds, apigenin-like signaling modulators.	Promotion of stress adaptation at modest stress intensity or autophagy-associated cell death under stronger perturbation.	p-AMPK, p-mTOR, ULK1 activation, LC3 dynamics, lysosomal blockade assays.	LC3 elevation without flux testing is insufficient to define the pathway outcome.	[4,23,28,33,66]
PI3K/AKT/mTOR survival signaling	PI3K, AKT, mTOR.	Particularly important in PTEN-deficient or stress-adapted disease states.	Genistein, quercetin, catechins, apigenin.	Reduced proliferative and survival signaling, with secondary effects on autophagy balance.	p-AKT, p-mTOR, proliferation markers, apoptosis markers, and autophagy flux assays.	Downstream autophagy is reported to remain weak unless lysosomal competence is assessed.	[5,23,66]
NF-kB/inflammatory redox signaling	NF-kB, cytokine-related inflammatory outputs, and oxidative stress amplifiers.	Relevant to inflammatory tone, tumor-microenvironment interaction, and obesity-associated signaling.	Catechins, isoflavones, apigenin, quercetin.	Reduction in inflammatory stress and possible reshaping of tumor-promoting microenvironmental cues.	NF-kB activation, inflammatory mediators, oxidative biomarkers, and paired phenotypic assays.	Lower inflammatory signaling does not necessarily translate into clinically meaningful interception.	[23,25,26,29,30,59,66]
TFEB/lysosomal competence/ferritinophagy	TFEB, lysosomal biogenesis, ferritin turnover, and iron release.	Important when flavonoids are proposed to affect lysosomal stress, ferritinophagy, or ferroptotic sensitivity.	Luteolin; selected catechins/EGCG as mechanistic precedents.	Increased lysosomal engagement, altered iron handling, and possible ferroptotic vulnerability.	TFEB localization, ferritin turnover, lysosomal markers, labile iron, lipid peroxidation, GPX4/SLC7A11.	Ferroptosis-related claims require dedicated readouts and should not be inferred from ROS alone.	[32,33,43,63,64]
Mitophagy and mitochondrial quality control	PINK1, Parkin, mitochondrial membrane integrity, ROS generation.	Relevant to bioenergetic adaptation and survival under chronic oxidative stress.	Most likely catechins and selected flavonols/cell stress modulators.	Improved mitochondrial quality at low stress, or mitochondrial control collapse under stronger challenge.	Mitochondrial membrane potential, mitophagy markers, mitochondrial ROS, and colocalization assays.	Global ROS reduction cannot substitute for mitochondrial quality-control analysis.	[23,28,32]

**Table 6:** Autophagy-associated states and candidate translational vulnerabilities arising from an organelle-centered interpretation.

Autophagy-Associated State	State-Based Interpretation	Minimum Evidence Required	Candidate Translational Vulnerability or Design Implication
Homeostatic control	Balanced autophagic flux with preserved lysosomal competence and controlled redox burden. Evidence is strongest when supported by localized disease or tissue-linked endpoints.	LC3 and p62/SQSTM1 dynamics, cargo turnover, lysosomal pH or proteolytic activity, and stage-appropriate tissue or pathology-linked endpoints.	Prioritizes prevention or surveillance-window designs and avoids interpreting homeostasis as direct tumor cytotoxicity.
Adaptive tumor survival	Active autophagy supports viable tumor-cell adaptation under hypoxia, androgen stress, therapy pressure, or metabolic strain.	Flux-aware assays combined with viability, rescue logic, p62/SQSTM1-NRF2 status, AR/PTEN/PI3K/AKT context, and pathway-specific readouts.	May reveal dependencies on lysosomal activity, NRF2 buffering, mitophagy, or mTOR-linked stress adaptation.
Blocked clearance	Autophagosome or p62/SQSTM1 accumulation reflects impaired lysosomal degradation rather than productive recycling.	Lysosomal pH, cathepsin activity, cargo degradation, pharmacologic flux blockade, and exclusion of CASM-related misclassification.	Supports testing lysosomal overload, clearance failure, or repair pathways as organelle-centered vulnerabilities.
Stress-overload vulnerability	Redox, iron, mitochondrial, or lysosomal stress exceeds the cell’s adaptive capacity, shifting it toward death-associated outputs.	Labile iron, lipid peroxidation, GPX4/SLC7A11, mitochondrial stress, ferroptosis/apoptosis rescue controls, and phenotype-level endpoints.	Supports combination strategies that intentionally push organelle stress beyond adaptive buffering capacity.

**Table 7:** Compound evidence-stratification matrix for interpreting flavonoid and non-flavonoid stress perturbations.

Compound Group	Evidence Anchor	Role in the Framework	Main Caution
Green tea catechins/EGCG	Tissue-exposure support: HGPIN, pre-prostatectomy, and tissue-exposure designs [45,54].	Benchmark for linking human exposure, tissue sampling, and flux-aware interpretation.	Tissue levels may be low; LC3/p62 changes still require flux and lysosomal validation.
Soy isoflavones/genistein/daidzein/equol	Tissue-exposure support. Pre-prostatectomy and metabolite-stratified evidence [46].	Model for host metabolic stratification and microbiome-dependent exposure.	Equol-producer status and tissue recovery must be predefined.
Quercetin	Human tissue concentration studies and the broad preclinical literature.	Useful for exposure-realism and static-marker-inflation critique.	Mechanistic richness can exceed evidence for autophagic flux.
Luteolin/apigenin	Mostly mechanistic and preclinical; luteolin links TFEB, iron handling, and ferritinophagy.	Probe for ferritinophagy/ferroptosis-aware interpretation.	Requires prostate-specific and localized-disease validation; luteolin background now cites [53]
Carnosic acid	Non-flavonoid phenolic diterpene with cancer autophagy/apoptosis literature.	Comparator showing framework applicability beyond flavonoids [52]	Currently supportive rather than localized prostate-confirmatory.
Curcumin/resveratrol	Preclinical prostate-cancer literature and broader polyphenol evidence.	Non-flavonoid comparators for stress-response and autophagy-state classification [55,56]	Bioavailability, formulation, and context-dependent autophagy remain limiting factors.

**Table 8:** Representative flavonoid-associated perturbation case studies used to stress-test autophagy interpretation in localized prostate cancer.

Flavonoid Class/Representative Compounds	Main Redox-Related Actions	Autophagy-Related Relevance	An Interpretive Setting Where Evidence Is Most Informative	Primary Interpretive Use	Main Limitation or Caveat	Representative References
Green tea catechins/EGCG	Modulate oxidative tone, inflammatory signaling, mitochondrial stress responses, and AR-related pathways.	Frequently linked to autophagy-associated phenotypes, including flux, p62/SQSTM1-NRF2 regulation, and lysosomal acidification; however, not all studies distinguish induction from blocked or cytoprotective flux.	HGPIN, pre-prostatectomy window studies, and tissue-exposure designs.	Benchmark for assessing how far human tissue sampling and controlled exposure can constrain claims about autophagy.	Tissue concentrations remain lower than those observed in many in vitro exposures, and interpretation is weakened when flux-aware autophagy assays are not performed.	[9,10,11,12,31,39,46,47,54,63,67,68,69]
Soy isoflavones/genistein, daidzein, equol	Influence redox signaling, endocrine crosstalk, NF-kB, Akt-related pathways, and inflammatory tone.	Mechanistically relevant, but effects are likely modified by hormonal context and microbial metabolism.	Pre-prostatectomy intervention, dietary supplementation, and metabolically heterogeneous cohorts.	Model for testing how metabolite identity and equol status shape the meaning of autophagy-related readouts.	Interindividual variation in equol production introduces major heterogeneity and may influence response.	[7,13,44,46,69,70]
Apigenin	Modulates NF-kB, STAT3, MAPK, and AR-linked signaling while affecting oxidative and inflammatory stress responses.	Has been linked to apoptosis and autophagy-related markers in experimental systems.	Mechanistic preclinical models in which the signaling context can be defined in advance.	Probe for determining when apoptosis- or autophagy-associated marker movement is being inferred rather than resolved.	Current evidence is mainly mechanistic and does not yet establish a clinically credible exposure-response framework in localized prostate cancer.	[66]
Luteolin	Alters oxidative tone and iron-related stress handling, with growing relevance to ferroptotic vulnerability.	Particularly relevant because it has been linked to TFEB nuclear translocation, ferritinophagy, and crosstalk between lysosomal biology and ferroptosis.	Stress-sensitive models in which iron handling, lysosomal status, and ferroptosis-aware controls can be measured together.	Probe for testing whether lysosomal engagement and ferritin turnover are being interpreted with sufficient mechanistic discipline.	Human evidence is still limited, and mechanistic interpretation requires stronger integration of lysosomal, iron, and lipid peroxidation readouts.	[32,33]
Quercetin and related flavanols	Reduce inflammatory signaling, suppress proliferation-related pathways, and modulate oxidative stress responses.	Often discussed in relation to autophagy-associated phenotypes, but many studies rely on static markers and supraphysiologic exposures.	Models in which static-marker inflation and exposure mismatch can be made visible.	Cautionary case for distinguishing mechanistic versatility from resolved autophagic meaning.	The quality of the literature is uneven, and claims are frequently weakened by inadequate pharmacologic and autophagy methods.	[23,27,28]
Carnosic acid/phenolic diterpene comparator	Modulates antioxidant defense and has been linked to apoptosis- and autophagy-related tumor-cell stress responses.	Useful as a non-flavonoid stress probe for testing whether the framework extends beyond flavonoids.	Preclinical and extra-prostate or broader cancer context.	Comparator for broader applicability of the flux-aware framework.	Localized prostate cancer validation and tissue exposure data remain limited.	[52]
Curcumin and resveratrol/non-flavonoid polyphenols	Affect PI3K/Akt/mTOR, NF-kB, AR-related signaling, calcium/ER stress, and autophagy-associated death or survival pathways.	Demonstrate that flux-aware state assignment applies to broader polyphenol stress responses.	Prostate preclinical models, formulation-focused literature, and broader cancer autophagy evidence.	Non-flavonoid examples for framework transferability.	Bioavailability, formulation heterogeneity, and context-dependent autophagy restrict direct translational claims.	[55,56]

Abbreviations: EGCG, epigallocatechin gallate.

**Table 9:** Human studies that help classify, rather than merely claim, autophagy-associated responses in localized prostate cancer.

Intervention/Compound	Clinical Setting	Main Finding	Interpretive Contribution	Main Limitation	Representative References
Green tea catechins	Men with HGPIN.	Reported reduction in prostate cancer incidence during follow-up in a prevention-oriented high-risk setting.	Shows how a human prevention signal can be discussed without assuming that the meaning of autophagy has already been resolved.	Small study context and need for broader confirmation using modern pharmacodynamic endpoints.	[9,10]
Polyphenon E	Pre-prostatectomy window trial in localized prostate cancer.	Demonstrated feasibility, controlled administration, and biological sampling opportunity before surgery.	Useful for linking oral intake to tissue-level sampling in localized disease, which is essential for interpreting autophagy claims.	Does not by itself define which autophagy or redox changes are clinically meaningful.	[12]
Genistein	Short-term intervention before radical prostatectomy.	Feasible and well-tolerated, with measurable exposure-related changes.	Supports the use of short pre-surgical studies to constrain exposure realism and pathway-level interpretation.	Biological heterogeneity and uncertain linkage between short-term biomarker shifts and longer-term benefit.	[13]
Soy/isoflavone supplementation	Randomized controlled trial literature summarized in meta-analytic form.	Suggests biological plausibility and safety, but no uniform clinical conclusion across trials.	Highlights why stratified designs are more informative than pooled claims about flavonoid effects.	Different formulations, doses, study durations, and endpoints reduce comparability.	[7]
Pomi-T whole-food polyphenol-rich supplement	Men with prostate cancer are monitored for PSA-related progression.	Supports the idea that complex polyphenol-rich interventions can produce measurable biological signals.	Demonstrates the interpretive limits of complex mixtures when several bioactive components move in parallel.	Mixture design limits attribution to specific flavonoids or mechanisms, including redox or autophagy effects.	[14]
Human prostate tissue exposure studies	Tissue-based pharmacology after intake of tea polyphenols, soy isoflavones, or resveratrol-related species.	Demonstrate that phytochemical-derived species can reach prostate tissue, often with chemical forms different from those used in vitro.	Provides the pharmacologic context needed to assess whether later claims about autophagy are chemically plausible.	Tissue concentrations remain low relative to many experimental conditions, and ex vivo deconjugation can distort interpretation.	[45,46,47,48,49]

**Table 10:** Main interpretive barriers and design corrections required for flux-aware studies in localized prostate cancer.

Translational Barrier	Why Does It Distort Interpretation	What Should Be Changed	Representative References
Use of supraphysiologic concentrations in vitro	Generates phenotypes that may not be achievable in human plasma or prostate tissue.	Prioritize exposure ranges supported by human pharmacokinetic and tissue distribution data.	[45,46,54]
Testing parent aglycones when human tissues contain mostly conjugated metabolites	Mechanistic conclusions may not reflect the chemical species that actually reach the prostate.	Incorporate conjugated metabolites, metabolite profiling, and chemically realistic exposure models.	[45,46,47,48,49,62]
Ex vivo deconjugation artifacts during tissue processing	Artificially inflates apparent aglycone levels and misrepresents tissue exposure.	Control sample handling rigorously and report processing conditions explicitly.	[48,49]
Reliance on LC3-II accumulation without flux testing	Cannot distinguish productive autophagy from impaired lysosomal clearance.	Use lysosomal inhibition, paired LC3 and p62/SQSTM1 interpretation, and orthogonal imaging or colocalization.	[24,57,58]
Insufficient attention to p62/SQSTM1-NRF2 and SPOP-related context	Misses a key interface between redox adaptation and autophagy rewiring in prostate cancer.	Incorporate pathway-specific biomarkers when mechanistic claims involve antioxidant adaptation or proteostasis.	[19,20,22,39,40]
Failure to stratify by equol-producer phenotype or microbiome-dependent metabolism	Biologically distinct responders may be pooled together, masking the signal.	Include equol status, microbial biotransformation context, or related stratification variables when studying isoflavones.	[44,70,74]
Ignoring obesity and the peri-prostatic adipose tissue context	Host metabolic status can reshape oxidative tone, inflammation, lipid handling, and tumor-promoting signaling.	Incorporate metabolic phenotype, adiposity-related biomarkers, and contextual interpretation of microenvironmental stress.	[59]
Lack of ferroptosis-aware endpoints in iron-related or TFEB-linked studies	ROS changes alone cannot establish ferroptotic vulnerability or ferritinophagy-driven phenotypes.	Measure lipid peroxidation, labile iron, GPX4/SLC7A11, and rescue with ferroptosis-relevant controls.	[32,42,43,77]
Weak or non-comparable clinical endpoints	Positive biomarker shifts cannot be integrated when studies use unrelated outcomes.	Use stage-appropriate and comparable endpoints, including tissue pharmacodynamics and clearly defined surveillance metrics.	[7,9,10,11,12,13,14]

## Data Availability

No new data were created or analyzed in this study.

## Abbreviations

The following abbreviations are used in this manuscript:

AR	Androgen receptor
ASAP	Atypical small acinar proliferation
CASM	Conjugation of ATG8 to single membranes
EGCG	Epigallocatechin gallate
GPX4	Glutathione peroxidase 4
HGPIN	High-grade prostatic intraepithelial neoplasia
LC3	Microtubule-associated protein 1 light chain 3
NCOA4	Nuclear receptor coactivator 4
NRF2	Nuclear factor erythroid 2-related factor 2
p62/SQSTM1	Sequestosome 1
ROS	Reactive oxygen species
SLC7A11	Solute carrier family 7 member 11
TFEB	Transcription factor EB
